# Experimental Quantification of Long Distance Dispersal Potential of Aquatic Snails in the Gut of Migratory Birds

**DOI:** 10.1371/journal.pone.0032292

**Published:** 2012-03-05

**Authors:** Casper H. A. van Leeuwen, Gerard van der Velde, Bart van Lith, Marcel Klaassen

**Affiliations:** 1 Department of Aquatic Ecology, Netherlands Institute of Ecology (NIOO-KNAW), Wageningen, The Netherlands; 2 Department of Aquatic Ecology and Environmental Biology, Institute for Water and Wetland Research, Radboud University Nijmegen, Nijmegen, The Netherlands; 3 Department of Animal Ecology and Ecophysiology, Institute for Water and Wetland Research, Radboud University Nijmegen, Nijmegen, The Netherlands; 4 Netherlands Centre for Biodiversity Naturalis, Leiden, The Netherlands; 5 Department of Animal Ecology, Netherlands Institute of Ecology (NIOO-KNAW), Wageningen, The Netherlands; 6 Centre for Integrative Ecology, Deakin University, Geelong, Australia; University of Hull, United Kingdom

## Abstract

Many plant seeds and invertebrates can survive passage through the digestive system of birds, which may lead to long distance dispersal (endozoochory) in case of prolonged retention by moving vectors. Endozoochorous dispersal by waterbirds has nowadays been documented for many aquatic plant seeds, algae and dormant life stages of aquatic invertebrates. Anecdotal information indicates that endozoochory is also possible for fully functional, active aquatic organisms, a phenomenon that we here address experimentally using aquatic snails. We fed four species of aquatic snails to mallards (*Anas platyrhynchos*), and monitored snail retrieval and survival over time. One of the snail species tested was found to survive passage through the digestive tract of mallards as fully functional adults. *Hydrobia (Peringia) ulvae* survived up to five hours in the digestive tract. This suggests a maximum potential transport distance of up to 300 km may be possible if these snails are taken by flying birds, although the actual dispersal distance greatly depends on additional factors such as the behavior of the vectors. We put forward that more organisms that acquired traits for survival in stochastic environments such as wetlands, but not specifically adapted for endozoochory, may be sufficiently equipped to successfully pass a bird's digestive system. This may be explained by a digestive trade-off in birds, which maximize their net energy intake rate rather than digestive efficiency, since higher efficiency comes with the cost of prolonged retention times and hence reduces food intake. The resulting lower digestive efficiency allows species like aquatic snails, and potentially other fully functional organisms without obvious dispersal adaptations, to be transported internally. Adopting this view, endozoochorous dispersal may be more common than up to now thought.

## Introduction

Widespread geographical ranges and fast colonization by aquatic organisms have fascinated biologists for a long time [1]–[3]. How can isolated wetlands, with varying water quality and short life spans, harbor a high biodiversity? A plausible explanation is long distance dispersal, whereby remote, new or only temporarily suitable wetlands are (repeatedly) colonized from a larger pool of biodiversity [4], [5]. However, this requires aquatic species to either disperse actively across land over long distances, or be suitable for passive transport by vectors such as wind (anemochory) [6], [7], water (hydrochory) [8] or other animals (zoochory) [9]–[11].

Waterbirds function as passive dispersal vectors for smaller organisms and are considered especially suitable because of their high abundances and directed flights between ecologically comparable habitats [12]–[14]. Birds have been caught while carrying seeds, algae and aquatic invertebrates between their feathers, on their bill or on their feet (ectozoochory) [15]–[18]. Additionally, birds have been found to carry viable aquatic organisms in their digestive system (endozoochory), which probably occurs at even higher frequency than external transport [17]. This indicates that not only parasites can survive in the digestive systems of animals, but also some free-living aquatic organisms [15], [19]–[24]. However, our taxonomic knowledge on which species are capable of surviving passage through the digestive system of waterbirds is still limited.

Most of the propagules recovered from droppings have so far been plant seeds or cryptobiotic life stages of aquatic invertebrates [25]–[27]. However, also some aquatic organisms have been retrieved from droppings while they were in fully functional, non-cryptobiotic life stages [28]–[33]. For these fully functional organisms we still lack knowledge on their actual dispersal potential, on which we focus here. Although their presence in droppings indicates that they can survive passage through the digestive tract, it still remains unknown whether or not they were retained in the digestive system long enough for dispersal over a significant distance. They might have been excreted shortly after ingestion, and thus their survival might contribute little to actual dispersal. For many of the cryptobiotic life stages found in droppings, their potential for long-distance dispersal has been assessed by combining field observations with experimental assessment of retention times and survival rates of the various propagules [34]. To date, we are aware of only one study addressing this for fully functional organisms, i.e. adult ostracods have been shown to survive long retentions in the digestive system of killdeer (*Charadrius vociferus*) [35].

We experimentally investigated the role of endozoochory for fully functional aquatic organisms when passing the digestive tract of waterbirds to assess its significance for long distance dispersal. We investigated this by determining the ability of aquatic snails (Gastropoda) to pass the digestive tract of mallards (*Anas platyrhynchos*). One snail species, *Hydrobia (Peringa) ulvae*, has previously been found to survive gut passage of shelducks (*Tadorna tadorna*) [28]. However, whether or not these snails were retained long enough for effective long distance dispersal remains unknown [36]. Endozoochory could be a plausible explanation for the widespread distributions and fast colonization of many aquatic snail species, and there are many invasive aquatic snails, such as *Physella (Haitia) acuta* [37], *Bithynia tentaculata* [38] and *Potamopyrgus antipodarum* [39] with unknown dispersal vectors. Since snails may be important vectors for parasites such as the trematodes *Fasciola* sp. (liver fluke) and *Microphallus* sp. that can harm waterbirds and cattle [40], it is also of applied relevance to know the dispersal capabilities of aquatic snails.

We performed two complementary experiments in which we tested both the survival potential and the associated retention times of four aquatic snail species after ingestion by mallards. The two experiments differed in emphasis. Experiment 1 tested the survival and potential dispersal distances of the four species, while experiment 2 concentrated on the two snail species with the highest potential of survival, for which faeces were sampled at a higher frequency and the number of mallards and snails was increased. To mimic accidental ingestion of invertebrates by herbivorous waterbirds, we added the simultaneous ingestion of macrophytes and snails to experiment 2. We hypothesize that aquatic snails can be retained long enough in the digestive system of mallards to be successfully dispersed over long distances.

## Materials and Methods

### Ethics statement

These experiments have been carried out under license numbers CL07.04 and CL08.02 of the Royal Netherlands Academy of Arts and Sciences (KNAW) animal ethics committee that specifically addressed our two experiments. No specific permits were required for the described field studies. All snails were sampled from public terrain. The locations were not protected and none of the sampled species is endangered or protected.

### Snails

Four snail species were chosen for the experiments, each with a widespread distribution throughout the Netherlands suggesting good dispersal capacities. With respect to their chance of surviving passage through the digestive tract we note that *Potamopyrgus antipodarum* is known as a successful invasive species [39] and has a wide tolerance to environmental conditions [41], [42]. *Bithynia leachii* was chosen as native species. *Hydrobia (Peringia) ulvae* is a marine species related to *P. antipodarum*, and known to survive digestion of shelducks [28], [33]. All these three prosobranch species posses an operculum, which is a calcareous or horny lid that can close the shell aperture. The fourth species was *Bathyomphalus contortus*, a common planorbid species that was included because of its different shell morphology (flat) compared to the other species. This pulmonate species does not have an operculum. Table S1 provides the sampling locations, shells sizes and further morphological information on the species.

All snails involved in the two experiments were collected a maximum of two days prior to their use in an experiment. They were kept in aquaria that we filled with water collected at their sampling locations, at a constant temperature of 15°C. Before each experiment a random subset of each species was measured for length and width to the nearest 0.1 mm with calipers. Thereby shell size was defined as the maximum measurable size of the shell (shell height in the case of the prosobranchs, and shell diameter in the case of the planorbid species) [41]. In the morning of each experimental day, the snails were taken from the aquaria and portions of 50 individuals were surrounded by a 1 to 2 mm layer of dough (i.e. moisturized grinded wheat seeds) to create pill-shaped “pellets” that facilitated feeding. We previously assessed 100% survival of snails in pellets over a period of 4 hrs (n = 50 per pellet, tested 2 pellets for each species), all snails thus entered the mallards in good condition as if they were swallowed simultaneously with a minor amount of grinded seeds.

### Experiments

The procedure during both experiments was to take the mallards from their outdoor aviary at 0800 hours on each experimental day. They were weighed and fed 100 to 300 snails depending on the treatment, and subsequently kept in individual hardboard cages (LWH: 0.54×0.46×0.48 m) for 24 hours. The birds had continuous access to water but not to food, resembling flying conditions as much as possible. The front of each cage was made of 12 mm mesh wire and the cages were placed side by side, so that the birds could see their surroundings but not each other. The floor was constructed of the same mesh wire, which allowed us to collect faeces in a removable tray without disturbing the birds. The removable trays were filled with filtered water from the snail species' sampling location to dissolve faeces immediately after excretion.

At regular intervals (depending on the experiment, see above) the content of the removable trays was sieved using a 0.5 mm mesh. Viability of snails was checked immediately upon retrieval by looking for movement or retraction reactions after touch under a microscope. If in doubt, survival was subsequently checked every four hours up to 48 hours after excretion, until ascribed to the categories “alive” or “dead”. Viability was monitored for three months after retrieval by keeping the viable snails in aquaria at 15°C. Shells without viable snails showing no visible damage, and of which length and width could be measured, were defined as ‘intact shells’. Broken shells and parts of shells were defined as ‘damaged shells’.

Experiment 1 was conducted between 5 and 27 September 2007. All four snail species were fed once to each of six male and six female mallards in a random block design. Due to low availability of *Bathyomphalus contortus* and *Bithynia leachii* we fed 100 individuals of these species to each of the 12 mallards, whereas for *P. antipodarum* and *H. ulvae* we fed 200 individuals per mallard, maximizing the effect of detecting potential survival. Faeces were sampled at 1, 2, 4, 8, 12 and 24 hours after feeding. Mallards were allowed one week of recovery in between experimental days.

Experiment 2 was conducted between 6 and 20 August 2008. Seven male and seven female mallards were used, of which four individuals had also been used in experiment 1. Each mallard was fed 300 *H. ulvae*, 300 *P. antipodarum*, or a mixture of 150 *P. antipodarum* and 1.0 gram fresh weight *Elodea nuttallii*. Feeding was done in a random block design over three weeks, again allowing mallards a one-week recovery between experiments. Faeces were sampled every hour for the first 12 hours, and once after 24 hours.

### Mallards

Mallards were chosen because they represent common omnivorous, migratory waterbirds with a widespread distribution [43]. Both freshwater and marine aquatic snails are part of their regular diet [44]–[47]. In addition, mallards are opportunistic feeders with their diet composition greatly determined by availability of the potential food items in their habitat [48]. They thus potentially ingest large amounts of similar propagules. For instance, up to 1200 snails of *P. antipodarum* were found per mallard shot in Ireland [49]. Mallards behave well in captivity and can be used with minimal stress during experiments. They are therefore suitable and frequently chosen for dispersal studies [27], [50].

All experimental mallards were of Dutch origin, captive bred and originally obtained from a waterfowl breeder (P. Kooy and Sons, ‘t Zand, The Netherlands). They had been housed in the outdoor aviary of the Netherlands Institute of Ecology in Heteren, The Netherlands, for at least 2 years prior to the experiments. They were kept on a stable diet of commercial pellets (Anseres 3®, Kasper Faunafood, Waalwijk, the Netherlands) and seed-based mixed grains (HAVENS Voeders ®, Maashees, Cary, NC, USA). One week prior to each experiment, the mallards were subjected to the experimental protocol to habituate them to the procedures and reduce stress. Male mallards (ranging from 1008 to 1288 g, with mean 1130±16 SD) were on average heavier than females (870 to 1155 g, mean 1001±22 SD, t = 5.7, df = 12, p<0.001).

### Statistical analyses

Because no intact shells were retrieved from *B. contortus*, and only 1.0% retrieval of shells from *B. leachii*, we concentrated statistical analyses on retrieval of intact *H. ulvae* and *P. antipodarum*. Where appropriate, data from both experiments were combined by calculating the retrieved snails per 4, 8 and 12 hours after ingestion. For each trial and sampling interval, the probability that *H. ulvae* and *P. antipodarum* were retrieved intact was used as the binomial dependent variable in a generalized mixed model with binomial error distribution and logit link function (Table S3). Fixed factors included in the best model were snail species, mallard gender and whether or not macrophytes were fed together with the snails. Individual mallard was included as random factor nested in fixed factor gender. Retention time, number of propagules fed and mallard body mass at the start of each experimental day were taken as covariates. Covariates were centered to allow interpretation of the estimates at mean values. After model selection based on AIC criteria (see Table S3), interactions left in the model were “macrophytes and retention time” and “mallard body mass and retention time”.

The effect of retention time on the size of excreted intact snails was tested using a general linear model with the normally distributed length of excreted snails depending on retention time as factor and gender and individual mallards as random factors, using only the more detailed data from experiment 2. Whether the average length of excreted snails was different from that of ingested snails was tested using an ANOVA with post-hoc Tukey's HSD. All calculations were performed in R for statistics [51].

## Results

**Figure 1:**
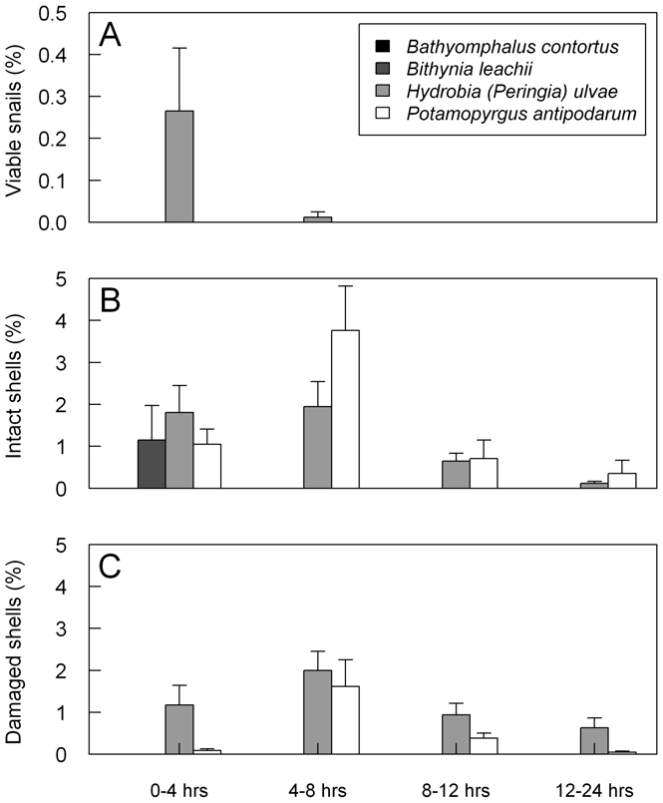
Percentage of ingested snails retrieved viable (A), intact (B) or damaged (C) as a function of retention time. Data for the two experiments combined.

Retrieval of viable snails and intact or damaged shells differed between snail species and changed with retention time (Table S2, Table S3, Fig. 1). Viable snails were retrieved up to five hours after feeding, but only for *H. ulvae* (Fig. 1A). Most viable snails of *H. ulvae* were retrieved in the first four hours after ingestion, and most intact shells of all snail species together between four and eight hours after ingestion (235 versus 366, respectively, Fig. 1B). Only 99 shells were retrieved between eight and 12 hours, and 26 shells between 12 and 24 hours after feeding (Fig. 1B). Birds with higher body mass excreted less intact snails, and this relation became more pronounced with increasing retention time (indicated by the negative interaction coefficient in a generalized mixed model, Table S3). Viable snails stayed alive for at least three months after retrieval.

The interaction between retention time and mallard body mass was the most important predictor in the model indicated by the highest standardized coefficient (Table S3). Based on the effect size of the interaction coefficient, but given large confidence intervals, an increase of body mass by 100 grams (our experimental birds ranged between 870 and 1288 g) would decrease the chance a bird excretes an intact snail by 3.9% at 4 hours after ingestion. The effect became more pronounced at longer retention times, with an increase of 100 g leading to a 22% and 37% reduced chance of intact snail retrieval at 8 and 12 hours after ingestion, respectively. Body mass was a more important predictor than mallard gender as indicated by the higher standardized coefficient in the model.

**Figure 2:**
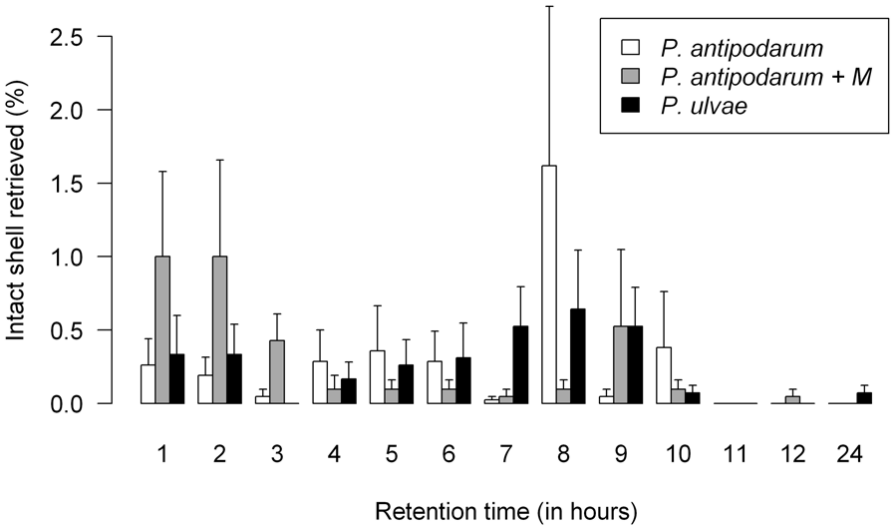
Percentage of ingested snails retrieved as intact shells (mean ± SE) as a function of snail species and retention time. “*P. antipodarum+M*” is feeding including macrophytes. Data from Experiment 2 exclusively.

**Figure 3:**
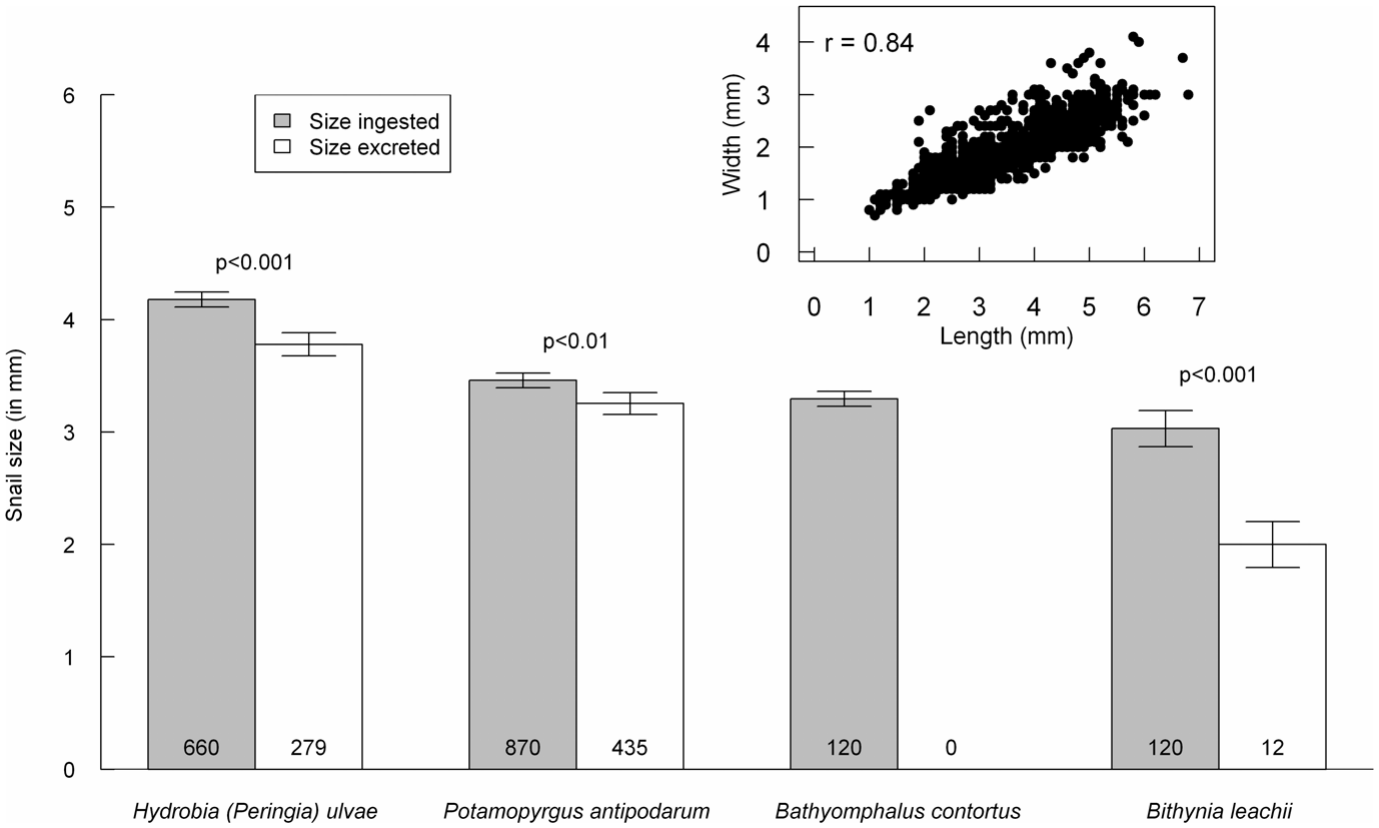
Average shell size of snails ingested and excreted for the four different species. Average shell size of excreted snails was smaller than the average shell size of ingested snails for all three species of which snails were retrieved (post-hoc Tukey's HSD, p-values indicated in the graph). Error bars indicate 95% CI of the mean and samples sizes are indicated at the bottom of the bars. Results for shell width were identical due to a strong correlation between shell length and width for all species (r = 0.84, p<0.001).

Addition of macrophytes to the feeding of the snails changed the release pattern of intact shells over time (indicated by the significant interaction with retention time in Table S3, and visualized in more detail in Fig. 2). The average size of excreted shells was smaller than that of ingested snails (both in terms of length and width) for all species (except for *B. contortus* where we did not retrieve any intact shells, Fig. 3). The size of retrieved intact shells did not differ with retention time (GLM, t = −1.21, df = 346, p = 0.22).

## Discussion

Our experiments showed that the aquatic snail *Hydrobia ulvae* is capable of surviving up to five hours in the digestive system of waterfowl. This indicates that not only cryptobiotic life stages, algae and plant seeds may use endozoochorous transport by surviving several hours in the digestive system of birds, but also fully functional, active life stages of invertebrates have endozoochorous dispersal potential.

*H. ulvae* has previously also been retrieved alive from shelduck droppings [28], [32], [33]. Our results now show that these snails may have originated from another location then where they were retrieved in droppings. Whereas many waterbirds can maintain sustainable flight speeds of up to 70 km h^−1^ [52], [53], a bird in straight flight might cover a distance of over 300 kilometers in five hours. Although the majority of snails that are potentially ingested before departure will be excreted within the first tens of kilometers [54], there is potential for birds to disperse snails over distances not easily achieved by the snails themselves. The actual dispersal distance and frequency will depend on more additional factors than could be included in our experiments, such as the timing and place of ingestion, behavior and activity of the birds during digestion, domestic or wild origin of the experimental birds, and bird species. Nevertheless, our results indicate there is potential for small operculated aquatic snails to survive prolonged digestion, which is an important requirement for successful dispersal.

Since travelling birds will generally not forage during actual moving we only provided water during the experiments. Continuous foraging during digestion has been shown to reduce digestive intensity [55], while fasting more likely improves digestive intensity by recirculation of food in the digestive system [56]. We choose to remove the food during the experiments, to estimate snail survival during the trials as conservatively as possible. To minimize stress of the mallards in the experiments we habituated them in a test trial the weeks before the start of each experiment. We rolled the snails in a thin layer of bread dough (grinded wheat seeds with some water) to minimize handling stress during feeding and allowing exact timing of ingestion. Starving the mallards before offering snails to make them forage voluntarily would not allow feeding of a known quantity of snails at a known time, which was required for estimating survival as well as retention times. We expected minimal influence of feeding by pellets, since each pellet contained only a minor amount of flour compared to the amount of snails. This resembles ingesting a minor amount of seeds simultaneous with the snails, which we also expect to occur in natural situations. This technique has frequently been applied successfully in previous studies on endozoochory [26], [27], [50], [57].

### Why do snails survive?

One potential explanation for survival of aquatic organisms is that transported organisms have evolved special adaptations for endozoochorous dispersal. This has been shown in many terrestrial seeds and fruits [58], [59]. For aquatic seeds, these adaptations have only been suggested more recently [27], and for aquatic invertebrates detected only sporadically [5]. While many resting stages of aquatic invertebrates are known to be adapted to survive temporarily unfavorable environmental conditions, to date it is unclear whether they are especially adapted for internal dispersal by birds.

Alternatively, such as in the case of aquatic snails, characteristics that make them suitable for internal dispersal may be attributed to adaptations likely acquired to survive normal environmental conditions. Both marine and freshwater habitats can be very dynamic, with fluctuating water levels, oxygen and nutrient concentrations and temperatures, requiring adaptations for survival. This requires comparable traits to the ones needed for endozoochory. Prosobranchs snails have a strong shell and operculum (a calcareous or horny lid). These characteristics probably evolved to protect them from predation and desiccation [60], but at the same time protect them from crushing forces in the gizzard and from digestive enzymes and juices entering the shell. Pulmonate snails lack these characteristics, and during our experiments, indeed no remnants of the pulmonate snail *B. contortus* were retrieved. The relatively weak shells of this species likely dissolved completely during digestion, while for the three operculated snails remains of their shells were retrieved. Malone [61] experimented with two other pulmonate snail species with relatively weak shells and without operculum, *Physa anatina* and *Helisoma trivolvis*. He also did not retrieve intact shells after feeding to birds. A strong shell thus facilitates both protection and survival during endozoochory. Additionally, the small size of many snail species may have several advantages in wetlands, such as survival during low food conditions, fast generation times in a stochastic environment or survival in small moist crevices. However, it is also an important trait of propagules for dispersal [27], [58], [62], and also led to increased probability of retrieval in our experiments (Fig. 3). The above mentioned examples illustrate that characteristics needed for successful dispersal by endozoochory are similar to those required for survival in stochastic environments such as wetlands.

This explanation for the survival of snails can be combined with an explanation involving the physiology of the birds as vectors. Generally, the efficiency with which birds digest their food is ∼75%, and can even be as low as 50% [63], [64]. An increase in digestive intensity will come with the cost of an increase in retention time [64], [65]. Hence, this may reduce food intake over time, at least in situations with high food availability. A maximum long-term average energy intake rate may thus be achieved at a less than 100% digestive intensity. This trade-off can provide a window of opportunity for all kinds of organisms to pass the digestive system undigested, even though they are not specially adapted for endozoochory.

### Significance of dispersal

The small percentage of the ingested snails that survived digestion raises the question of its significance for snail populations. However, due to the low energy content of one snail compared to the energy requirements of one water bird [66], [67], waterbirds can ingest large amounts of snails. Even low survival frequencies can lead to many individuals surviving. Many duck species are opportunistic feeders that ingest much of the same food source once abundantly available [43]. Shelducks have been estimated to ingest up to 33 000 *H. ulvae* per day [28], [32], and up to 1200 individuals of *P. antipodarum* have been collected per mallard in Ireland [49]. Illustrative, the shelduck droppings in which *H. ulvae* snails were retrieved in the field contained multiple viable snails per dropping [33].

However, what is the significance of endozoochorous dispersal for snails such as *H. ulvae*? Aquatic snails may also be transported by other vectors (reviewed by [12]), or externally by adhering to the outside of waterbirds (although evidence for “ectozoochory” by snails is still very limited [68] see also [36], [61]). Furthermore, *H. ulvae* is a marine snail that produces free swimming pelagic larvae, can float attached to the water surface [69] and has the capability to raft on drifting wood or plants. In contrast to freshwater snails, populations seem less dependent on long distance dispersal. Nevertheless, given the numerous waterbirds that forage on aquatic snails, even low frequency endozoochory may provide a constant dispersal mechanism connecting (marine) populations, and may connect different populations than other vectors. Marine populations have been shown to be (genetically) separated by ecological barriers in the sea [70], thus long-distance dispersal may enable range expansions along coastlines with more and less suitable sections, strong outgoing currents of rivers, or connect populations of coastlines separated by land or open water.

The fact that *P. antipodarum*, the freshwater snail closest related to *H. ulvae*, did not survive digestion indicates freshwater snail endozoochory might be less plausible than that of *H. ulvae*. Despite that *P. antipodarum* is such an effective invasive species [39], [42] for which dispersal by waterbirds between freshwater habitats may be even more relevant than for *H. ulvae*, it did not survive digestion of mallards in the quantities we offered. Based on our experiments, the success of *P. antipodarum* as an invasive species at this point cannot be attributed to birds as dispersal vectors, but is more likely caused by its other characteristics [41]. Nevertheless, very recently, a terrestrial snail species was found to survive digestion of passerine birds [71]. This indicates that more snail species than *H. ulvae* may be capable of endozoochory, including other fully functional aquatic organisms.

### Vectors

Mallards in this experiment represent omnivorous, common waterbirds with a highly variable diet including aquatic snails [45]–[47], [72]. Extrapolating our results to a field situation with other vector species has to be done conservatively. Although different species of *Anas* have shown similar capacity to disperse aquatic propagules [18], [73], [74], marked interspecific differences have also been found [19], [34], [75]. Nevertheless, besides surviving mallard digestion, *H. ulvae* is also capable of surviving shelduck digestion [33]. Specific experiments will be necessary to assess the dispersal potential of each vector-propagule pair separately, but the survival of snails in both shelducks and mallards strengthens the idea that potentially more (duck) species are capable of passing viable aquatic snails through their digestive systems.

More intact snail shells were retrieved from smaller mallards (Table S3), which may be due to smaller gut or gizzard sizes in smaller birds [20], [76]. Given that gut length and gizzard size are generally correlated to body mass [77], we expect smaller individuals to have shorter retention times leading to retrieval of more intact propagules. The effect of body mass became more pronounced with increasing retention time, although the large confidence intervals should be kept in mind. This suggests smaller mallards may not only marginally excrete more intact propagules, they may also continue to do so at longer distance from the location of ingestion.

### The effect of adding macrophytes

Digestive intensity is known to vary with the quality and type of food ingested [78]. Therefore we also tested snail survival and retention times when snails were ingested simultaneously with macrophytes. This resembles the field situation of accidental ingestion of invertebrates by herbivores, or a mixed diet by omnivores. Retrieval of intact snail shells was accelerated due to the addition of macrophytes to the diet (Fig. 2 and Table S3). This is in accordance with previous observations where retention times of brine shrimp eggs (*Artemia salina*) decreased when ingested with macrophytes [79]. A general decrease of viability with retention time observed for propagules [50], [73], [74] suggests that aquatic snails ingested with macrophytes will have increased survival chances, but shorter dispersal distances. Herbivorous birds that ingest invertebrates accidentally and omnivores that forage on invertebrates with macrophytes, may thus contribute to dispersal of invertebrates in natural situations. How this depends on macrophyte species, bird species and other parameters remain interesting avenues for future research.

### Conclusions

We have shown that the aquatic snail *H. ulvae* can survive long enough in the digestive tract of birds to potentially be dispersed over significant distances. We suggest this is possible with the adaptations this snail already acquired for surviving unfavorable circumstances in their natural habitat. We put forward that a digestive trade-off in birds makes endozoochory possible for propagules without special adaptations for endozoochory. The fact that besides cryptobiotic life stages of invertebrates, algae and plant seeds also aquatic snails, as fully functional free-living aquatic organisms, can successfully be dispersed in the digestive system of birds suggests endozoochory is a more common mode of transport than currently realized.

## Supporting Information

Table S1 **Snail species used in the experiments.**
(PDF)

Table S2 **The number of intact, damaged and viable snails retrieved after 24 hours.**
(PDF)

Table S3 **Results of the generalized mixed model for the chance of retrieval of intact shells.**
(PDF)
