# Supplementary material for: Experimental Quantification of Long Distance Dispersal Potential of Aquatic Snails in the Gut of Migratory Birds
Source: PLoS One. 2012 Mar 5;7(3):e32292. doi: 10.1371/journal.pone.0032292 (PMC3293790; doi:10.1371/journal.pone.0032292)
Supplement: Table S1 — Snail species used in the experiments. (PDF) [file pone.0032292.s001.pdf]

Experimental quantification of long distance dispersal potential of aquatic snails in the gut of migratory birds

Casper H.A. van Leeuwen, Gerard van der Velde, Bart van Lith & Marcel Klaassen

Supporting Information

Table S1: Snail species used in the experiments.

Snail species	Family; Order	Respiratory system	Operculum	Sampling location (The Netherlands)	Size in mm (Average ± SD)	Notes
Bithynia leachii	Bithyniidae; Prosobranchia	gill	yes	Vechten (ditch) 52°03'33 N 05°10'14 E	L 2.9 ± 0.9 W 2.1 ± 0.6	strong, horn-shaped shell
Hydrobia (Peringia) ulvae	Hydrobiidae; Prosobranchia	gill	yes	Paesens (coast) 53°24'19 N 06°05'14 E	L 4.0 ± 0.8 W 2.1 ± 0.4	horn-shaped, marine
Potamopyrgus antipodarum	Hydrobiidae; Prosobranchia	gill	yes	Ooijpolder (lake) 51°51'12 N 05°53'18 E	L 3.4 ± 0.9 W 1.8 ± 0.4	horn-shaped, invasive
Bathyomphalus contortus	Planorbidae; Pulmonata	lungs	No	Loosdrecht (ditch) 52°09'42 N 05°01'52 E	L 3.3 ± 0.3 W 1.7 ± 0.2	flat, round shaped
