# Supplementary material for: Experimental Quantification of Long Distance Dispersal Potential of Aquatic Snails in the Gut of Migratory Birds
Source: PLoS One. 2012 Mar 5;7(3):e32292. doi: 10.1371/journal.pone.0032292 (PMC3293790; doi:10.1371/journal.pone.0032292)
Supplement: Table S2 — The number of intact, damaged and viable snails retrieved after 24 hours. (PDF) [file pone.0032292.s002.pdf]

Experimental quantification of long distance dispersal potential of aquatic snails in the gut of migratory birds

Casper H.A. van Leeuwen, Gerard van der Velde, Bart van Lith & Marcel Klaassen

Supporting Information

Table S2: The number of intact, damaged and viable snails retrieved after 24 hours for the two experiments combined.

**Species**	**Snails ingested**	**Damaged shells** (average ± 95%CI)	**Intact shells** (average ± 95%CI)	**Viable snails** (average ± 95%CI)
Hydrobia (Peringia) ulvae	12×200 + 14×300 = 6600	11.4 (±7.0): 4.33%	11.2 (±8.5): 4.23%	0.8 (±1.2): 0.32%
Potamopyrgus antipodarum	12×200 + 14×300 = 6600	4.8 (±3.6): 1.88%	13.9 (±10.7): 5.47%	-
Potamopyrgus antipodarum + macrophytes	14×150 = 2100	0.8 (±0.5): 0.52%	5.3 (±3.7): 3.52%	-
Bithynia leachii	12×100 = 1200	-	1.0 (±1.3): 1.0%	-
Bathyomphalus contortus	12×100 = 1200	-	-	-
