# Supplementary material for: Experimental Quantification of Long Distance Dispersal Potential of Aquatic Snails in the Gut of Migratory Birds
Source: PLoS One. 2012 Mar 5;7(3):e32292. doi: 10.1371/journal.pone.0032292 (PMC3293790; doi:10.1371/journal.pone.0032292)
Supplement: Table S3 — Results of the generalized mixed model for the chance of retrieval of intact shells. (PDF) [file pone.0032292.s003.pdf]

Experimental quantification of long distance dispersal potential of aquatic snails in the gut of migratory birds

Casper H.A. van Leeuwen, Gerard van der Velde, Bart van Lith & Marcel Klaassen

Supporting Information

Table S3: Results of the generalized mixed model for the probability of retrieval of intact shells. Significant and marginally significant factors are in bold. Repeatability for random factor mallard was 16.5%. Standardized coefficients were calculated to indicate the relative contribution of the different factors (including binomial factors) to the model. Initial AIC of the full model including “experiment” and all second order interactions was 278.1. After model selection and removal of insignificant interactions and insignificant “experiment”, AIC was reduced to 260.8 with a delta AIC of 2.7 to the next best model.

	**St. coefficients**	**SE coef**	**Z-value**	**Pr(>|z|)**
(Intercept)	- 0.22	0.42	0.87	0.39
Snail species	- 0.61	0.36	- 1.73	0.08
Mallard gender	- 0.053	0.60	- 0.088	0.93
Macrophytes	- 0.70	0.68	- 1.28	0.20
Retention time	- 0.53	0.055	- 0.37	0.71
Number of snails fed	- 1.04	0.0042	- 1.92	**0.055**
Mallard body mass	- 1.56	0.0036	- 2.54	**< 0.05**
Retention time : Mallard body mass	- 2.10	0.00062	- 3.00	**< 0.01**
Retention time : Macrophytes	- 1.56	0.13	- 2.25	**< 0.05**
